# X-ray imaging detectors for synchrotron and XFEL sources

**DOI:** 10.1107/S205225251500010X

**Published:** 2015-04-10

**Authors:** Takaki Hatsui, Heinz Graafsma

**Affiliations:** a RIKEN SPring-8 Center, RIKEN, 1-1, Koto, Sayo, Hyogo 679-5148, Japan; b Photon-Science Detector Group, DESY, Hamburg, Germany; c Mid-Sweden University, Sundsvall, Sweden

**Keywords:** X-ray imaging detectors, hybrid detectors, monolithic detectors, recent detector developments

## Abstract

Current trends for X-ray imaging detectors based on hybrid and monolithic detector technologies are reviewed. Hybrid detectors with photon-counting pixels have proven to be very powerful tools at synchrotrons. Recent developments continue to improve their performance, especially for higher spatial resolution at higher count rates with higher frame rates. Recent developments for X-ray free-electron laser (XFEL) experiments provide high-frame-rate integrating detectors with both high sensitivity and high peak signal. Similar performance improvements are sought in monolithic detectors. The monolithic approach also offers a lower noise floor, which is required for the detection of soft X-ray photons. The link between technology development and detector performance is described briefly in the context of potential future capabilities for X-ray imaging detectors.

## Abstract 2

Hybrid and monolithic detectors for X-ray imaging are reviewed.

## 1. Introduction   

In the late 1990s, developments began on dedicated X-ray imaging detectors for synchrotron radiation based on the direct conversion of X-rays within semiconductors. These resulted in experimental methodologies with higher accuracy and higher efficiency, and also paved the way for new types of experiment. Today, this type of detector has become a necessity in many fields of synchrotron radiation and laboratory X-ray sources. A strong push for dedicated detector development came with the birth of hard X-ray free-electron laser (XFEL) sources (Emma *et al.*, 2010 ▶; Ishikawa *et al.*, 2012 ▶; Abela *et al.*, 2006 ▶). In this *Feature article*, we report the current trends in X-ray imaging using two detector technologies, hybrid and monolithic detectors. Both technologies enable the imaging of X-ray patterns with unique capabilities, such as single-photon detection, high dynamic ranges and sharp images. In addition, these detectors are equipped with advanced microelectronics circuitry which enables faster frame rates, as shown by some systems providing over 10 000 frames per second. We discuss hybrid detectors first, followed by monolithic detectors. In each section, a selected number of detector systems are presented. However, the examples do not indicate any relative importance, but simply align with the theme we discuss.

## 2. Interconnection methods: hybrid, monolithic and three-dimensional integration   

In solid-state X-ray detectors, the detection process can be split into two distinct steps. First, an incoming X-ray is absorbed by a photodiode and its energy transferred into electron–hole pairs *via* ionization. The electrons and holes subsequently drift in opposite directions under the influence of an electric field. In the second step, the signal generated by the drifting charges is measured and processed by the readout electronics. X-ray imaging detectors generally have interconnections between the photodiode and the signal-processing circuitry, but currently none of the interconnection methods meets all the requirements of the X-ray imaging detector. In this review, we discuss the detector project in terms of the interconnection method, as it has a critical impact on the detector capability.

**Figure 1:**
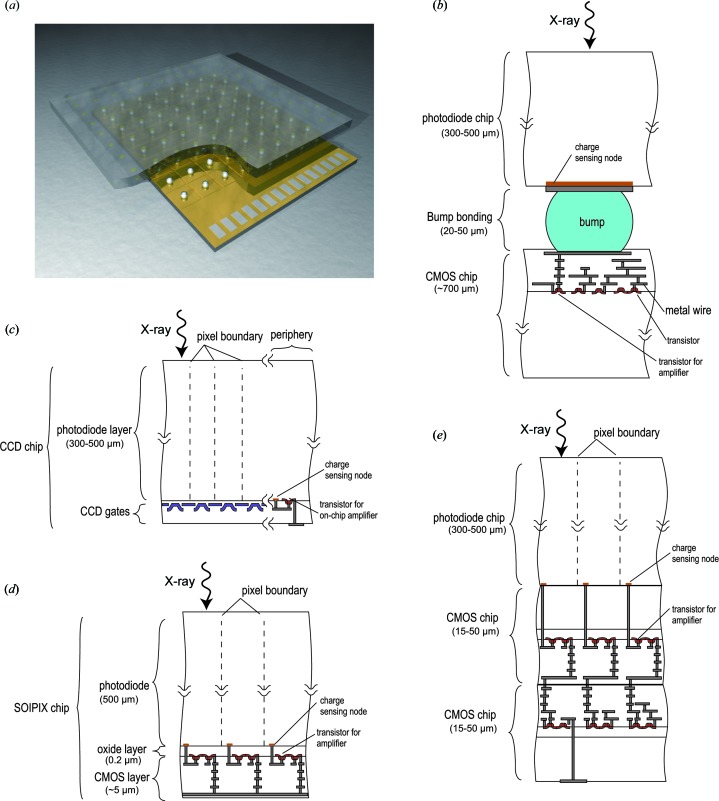
(*a*) Schematic layout of a hybrid detector. The top layer is the pixelated sensor, where the impinging X-ray photons are absorbed and their energy transferred into an electrical signal. Each pixel of the sensor is connected to a pixel of the readout chip, where the signal is further processed and transmitted to the backend electronics. (*b*) A cross-sectional view of a hybrid-detector pixel. The sensor, with a photodiode, and the readout chip are connected by a bump. The signal charge generated in the photodiode flows to the charge-sensing node and spreads over the bump and the transistor through metal wires, which have a large total capacitance. The resulting signal voltage is low and it is amplified by circuitry composed of transistors (red) in the readout chip. Because the photodiode and CMOS readout chips are fabricated separately, they can be optimized independently. (*c*) In contrast, charge-coupled device (CCD) detectors are made of a single chip. The pixel has a gate structure without transistors, which makes smaller pixels possible. The signal charge generated at the photodiode is transferred to the periphery of the sensor, where the charge-sensing node (orange) is located. The charge-sensing node is connected to the transistor (red). Because the charge is spread over a confined space, the associated capacitance is low and the resulting signal voltage becomes higher than that for a hybrid detector. Owing to this conversion scheme with a low input capacitance, noise performance as low as a few electrons is possible (see §4.1.1). (*d*) In SOI pixel technology (§4.2.1), the transistor for the amplifier is located inside the pixel, while the charge-sensing node and transistor are very close (less than 1 µm). The associated input capacitance is thus kept low, enabling better noise performance. However, the chip is made from a single SOI wafer, so optimization of the photodiode and CMOS transistor should be done in one production recipe. (*e*) For the VIPIC detector as an example of the three-dimensional integration case (§4.2.4), the charge-sensing node is located close enough to the amplifier transistor to provide a chance of realising low-noise performance. In addition, the photodiode and two CMOS chips can be optimized separately, giving considerable freedom in design and production. Thicknesses shown in this figure are ones for possible implementation and are provided here to give the reader an idea of physical size.

In a hybrid detector, the absorption and signal-processing processes are performed by two separate pieces of material which are connected together by high-density interconnects, most often bump-bonding. This is indicated schematically in Figs. 1 ▶(*a*) and 1 ▶(*b*). The advantage of this technology is that the absorption and signal processing can be optimized independently, providing greater flexibility. The disadvantage is the need for a fine-pitch or high-density interconnection between the two layers, which is a delicate, time-consuming and often expensive step. This also limits the smallest pixel size obtainable.

In contrast with hybrid detectors, there are other types of detector having both a photodiode for absorption and readout microelectronics for signal processing on a single chip. We define these as monolithic detectors. Modern microelectronics are fabricated on silicon and therefore the absorption material for monolithic detectors is, in practical terms, limited to silicon. As a consequence, the detection efficiency starts to drop at a photon energy range of 8–15 keV. Nevertheless, monolithic detectors offer unique advantages, such as low noise of less than three electrons and small pixels of less than 20 µm, which are not yet realised by hybrid detector technology (Figs. 1 ▶ *b*, 1 ▶ *c* and 1 ▶ *d*). The interconnection between the sensor and readout electronics fabricated by state-of-art microelectronics technologies offers several engineering benefits: a high yield of good pixels, uniform response, stable production and lower cost. Recently, the adoption of a new class of interconnection technologies, namely three-dimensional integration, has been reported and this is discussed in the last part of this review (Fig. 1 ▶ *e*).

## 3. Hybrid detectors   

X-ray imaging detectors come in two types, either photon-counting or integrating. In photon-counting systems, the signal generated by each absorbed photon is immediately processed and compared with user-defined thresholds in order to decide whether the observed signal corresponds to that expected from a photon of a certain energy. The number of photons passing the threshold criterion during the integration time is stored in a counter inside each pixel of the detector. A great advantage of photon-counting systems is that the processing electronics distinguish the signal generated by a photon of the desired energy against signals from photons of too high (higher harmonics) or too low (fluorescence) energy, as well as against the electronic background. This allows practically zero-noise performance. One example is the well known Pilatus detector (Eikenberry *et al.*, 2003 ▶) developed by the Swiss Light Source (SLS) at the Paul Scherrer Institute (PSI, Villigen, Switzerland) and commercialized by the company DECTRIS. A disadvantage of photon-counting systems is that they cannot handle large instantaneous fluxes. On the other hand, photon-integrating systems integrate the total signal, including noise, during a user-selected time. The advantage is that these systems can record very large instantaneous fluxes. This is mandatory for XFEL sources, but can also be advantageous for certain storage-ring based experiments. The disadvantage is that, for longer integration times, the noise generated by dark current can be significant. There is also no discrimination between photons of different energies.

We will describe current trends and developments, first for photon-counting systems and then for integrating systems. Subsequently, we will report on developments of high-*Z* sensor materials.

### 3.1. Current trends in photon-counting hybrid systems   

As stated above, various photon-counting hybrid systems have been introduced at synchrotron sources in recent years, including the Maxipix (Ponchut *et al.*, 2011 ▶), the XPAD (Delpierre *et al.*, 2001 ▶) and the well known Pilatus detector. It is difficult to overstate the success and impact of the Pilatus family of detectors on science at storage-ring sources. Nevertheless, the relatively large pixel size of 172 µm and the limited frame rate are now becoming bottlenecks in various experiments. As a follow-up to the Pilatus detector, the detector group at PSI have developed the EIGER system with a 75 µm pixel size, a reduction in pixel area of a factor of 5.26 (Dinapoli *et al.*, 2011 ▶). Each pixel contains a 12-bit counter and a 12-bit memory, which makes it possible to have a near dead-time-free readout; by storing the previous image in memory while it is being read out, the counter can start acquiring the next image almost immediately. Depending on the counter depth used, various frame rates are available, ranging from 8 kHz for a 12-bit counter depth to 23 kHz for 4 bits. The first systems have recently been installed on beamlines at various facilities around the world. The EIGER system will also be commercialized by DECTRIS and made available to the international community.

**Figure 2:**
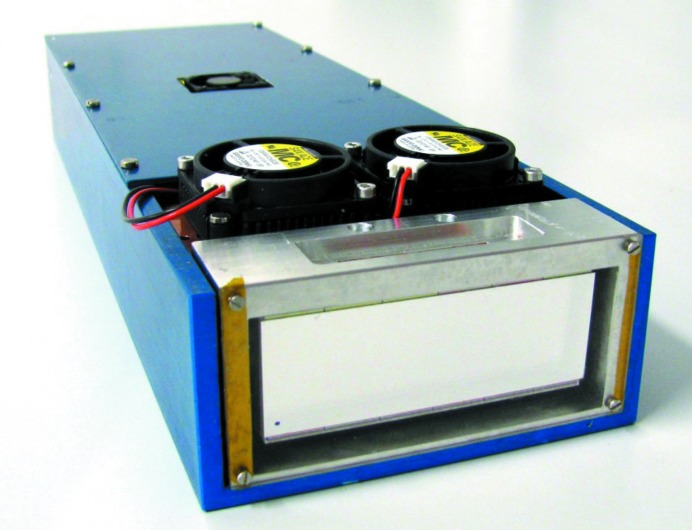
The LAMBDA system, an example of a detector system with a Medipix3 readout chip.

Similar progress towards smaller pixels and higher frame rates has been achieved by Medipix3-based systems (Ballabriga *et al.*, 2013 ▶). These have a 55 µm pixel size, which is a reduction in pixel area of a factor of nearly ten compared with the Pilatus detector, and close to a factor of two compared with EIGER systems. The Medipix3 also features two 12-bit counters per pixel, which can be used in a variety of ways: either switching between the counters to give a dead-time free readout, combining the two into a single 24-bit counter, or acquiring two images at different threshold settings simultaneously. With a 12-bit counter depth a frame rate of 2 kHz is achieved, and 24 kHz is possible for single-bit counter depths. One of the most innovative features of the Medipix3 readout chip is its communication between pixels. The principle idea is that, whenever a pixel detects a signal above a pre-set threshold, it ‘communicates’ with the surrounding pixels, and the charge that is spread over multiple pixels is summed together. This charge-summing mode overcomes the charge-sharing induced degradation of the energy resolution that is typical for small-pixel hybrid detectors. This is particularly important for systems using high-*Z* sensors like GaAs and CdTe, where fluorescence of the sensor plays a major role. Additionally, it is possible to use sensors of 110 µm pixel size and assign the incoming X-ray photons to eight different energy bins, providing coarse energy-resolving capabilities. This is particularly interesting for medical imaging applications, but might also find interesting uses at synchrotron sources. Various larger systems based on Medipix3 readout chips have been and are under construction, notably the Excalibur system at Diamond Light Source (Didcot, UK; Marchal *et al.*, 2013 ▶) and the LAMBDA system at DESY (Hamburg, Germany; Pennicard, Lange *et al.*, 2013 ▶), which is depicted in Fig. 2 ▶.

### 3.2. Current trends in photon-integrating hybrid systems   

The low-noise performance of photon-counting hybrid systems has proven to be a major advantage over systems with phosphor-coupled charge-coupled devices (CCDs) in many experiments, particularly in protein crystallography. However, a disadvantage of these systems is that photons are counted or treated one at a time, limiting the maximum flux that can be handled at storage-ring sources and completely excluding these systems from XFEL applications, where many photons per pixel arrive within a single pulse of less than 100 fs. Several dedicated developments have been initiated to address the formidable challenges imposed by XFELs. A few hybrid-detector development projects will be briefly described here.

#### 3.2.1. CSPAD and the ePix platform at LCLS   

The first XFEL-specific hybrid system to become operational was the CSPAD (Cornell–Stanford pixel array detector) developed for the Linear Coherent Light Source (LCLS) at the SLAC National Accelerator Laboratory (Stanford, California, USA; Philipp *et al.*, 2011 ▶). The LCLS delivers ultra-intense ultra-short X-ray pulses with a repetition rate of 120 Hz. Each single pulse is intense enough to produce a complete scattering pattern in the image, ranging from a single or no photons to 10^4^ or more photons per pixel. This requires both low-noise performance (in order to distinguish single photons) and a very high peak signal. The CSPAD has a pixel size of 110 µm and, when operated in high-gain (low-noise) mode, an equivalent noise of 1.1 keV. (Note that the magnitude of noise cited in this review is defined as standard deviation.) This mode provides a signal-to-noise ratio of 7 for 8 keV photons, which is sufficient to discriminate between individual photons. In this mode, a maximum of 350 photons can be detected per pixel. The system can also be operated in low-gain (high peak signal) mode, in which case up to 2500 photons of 8 keV can be detected per pixel with a noise floor of 3.5 keV. Since low-noise and high-peak signal modes cannot be used simultaneously, the user has to preselect the mode to be used. Various multi-megapixel systems have been produced and CSPAD detectors are used in most of the experiments performed at the X-ray pump–probe (XPP), coherent X-ray imaging (CXI), X-ray correlation spectroscopy (XCS) and matter in extreme conditions (MEC) stations, producing ground-breaking scientific results. With the experienced gained over the last few years, the science studied at XFELs has evolved rapidly and new experiments are being performed or planned. With that, the demands on the detector have changed, and the current CSPAD does not fulfil all the new requirements. In order to cater for these new experiments, the detector group at LCLS has started the design of a new generation of readout chips, called the ePix platform (Dragone *et al.*, 2014 ▶). The first member of this new family is the ePix100, with a pixel size of 50 µm, equivalent noise of 225 eV and a maximum detection limit of 100 photons per pixel at 8 keV. This system was specifically designed with X-ray photon correlation spectroscopy (XPCS) in mind. The second member under development is the ePix10k system, with 100 µm pixels, a peak signal of up to 10^4^ photons at 8 keV and an equivalent noise of 650 eV. A large peak signal with single-photon sensitivity is achieved by dynamic gain switching, which is described in more detail below. In parallel, the Cornell group has developed and is developing new detectors for high-speed imaging at both storage rings and XFEL sources, notably the MM-PAD and Keck-PAD systems (Tate *et al.*, 2013 ▶, Koerner & Gruner, 2011 ▶).

#### 3.2.2. LPD, DSSC and AGIPD detectors for the European XFEL   

The European XFEL currently under construction in Hamburg, Germany, presents an additional challenge for detectors. The European XFEL uses superconducting accelerators, permitting a very high bunch-repetition rate of up to 4.5 MHz during short burst periods of 0.6 ms, creating pulse trains with 2700 X-ray pulses. These bursts are then followed by 99.4 ms without bunches, resulting in a 10 Hz overall repetition rate. Since every pulse produces a complete diffraction pattern, and since 222 ns is too short to read out the million-pixel imaging detectors, the images have to be stored inside the pixels during the pulse trains and read out between pulse trains. In order to meet these challenges, three separate development projects have been funded, each using a different approach. The large-pixel detector (LPD) project uses three parallel gains to cover the high peak signal, and three associated analogue storage memories for storing up to 512 images during the pulse trains (Koch *et al.*, 2013 ▶). In order to incorporate these three independent detection chains, the pixel size has to be relatively large at 500 µm. The analogue data are converted to digital by on-chip ADCs (analogue-to-digital converters) and streamed off to the data backend for further processing between pulse trains. The LPD system is optimized for the highest flux experiments like liquid scattering, where spatial resolution is not critical. The DEPFET sensor with signal compression (DSSC) project uses a non-linear DEPFET (depleted *p*-channel field effect transistor) as the sensor, in order to cover the full range of intensity, and an in-pixel ADC plus a digital memory for storing the images (Porro *et al.*, 2012 ▶). Since digital memories are much more efficient, up to 800 images can be stored in the hexagonal pixels of approximately 200 µm. The DSSC system has the lowest noise and is optimized for the lowest-energy experiments at the SASE-3 beamline. The third project is the adaptive gain integrating pixel detector system (AGIPD; Henrich *et al.*, 2011 ▶), which uses a dynamically adapted gain, where each pixel automatically adapts its gain to the incoming number of photons, plus an analogue storage memory. The data are digitized off-chip between pulse trains. The 200 µm pixel size allows up to 352 images to be stored. The AGIPD system, with an equivalent noise of 1 keV, is optimized for general diffraction and imaging experiments between 5 and 25 keV, where single-photon sensitivity and a large peak signal of more than 10^4^ are required.

**Figure 3:**
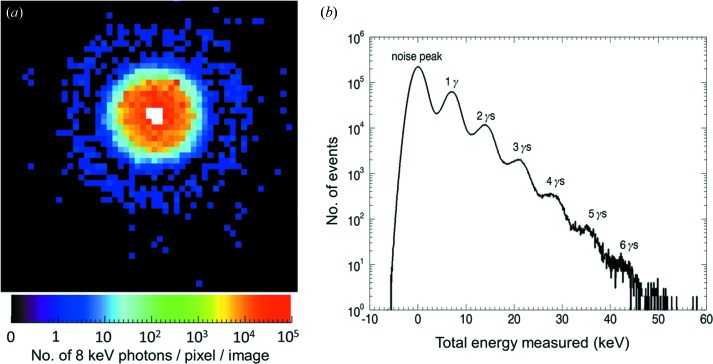
(*a*) An image of the direct beam and small-angle scattering region on the P10 beamline at PETRA III. (*b*) A histogram of the energy deposition in a pixel in the low-intensity region.

These performance characteristics are useful not only for FEL experiments but also for storage-ring stations, as shown in the example below. An AGIPD single-chip system with 64 × 64 pixels was tested on the DESY PETRA III P10 beamline in a small-angle scattering experiment using 500 nm spherical particles and an 8 keV photon beam. Since the system is designed to handle very high fluxes of more than 10^4^ photons per pixel per pulse, there was no need to use a primary beamstop. By accumulating 352 frames, which can be done at 4.5 MHz, and summing them together, the image shown in Fig. 3 ▶(*a*) was obtained. Note that the white pixels in the centre correspond to more than 10^5^ photons. At the same time, single-photon sensitivity was obtained for low-intensity regions. Fig. 3 ▶(*b*) shows a histogram of a pixel in the low-intensity region. Individual photons can be clearly identified and separated from the electronic noise.

This combination of single-photon sensitivity, 10^4^ photon peak signal and a 4.5 MHz frame rate will very likely also find new applications at synchrotron sources.

#### 3.2.3. Jungfrau and Mönch detectors at PSI   

The detector group at PSI, which is one of the partners in the AGIPD project, used the same adaptive gain switching concept in their Jungfrau detector for the SwissFEL (Mozzanica *et al.*, 2014 ▶). Since the SwissFEL will operate at 100 Hz, there is no need for in-pixel frame storage. A 75 µm pixel size was chosen, which seems well matched with most of the planned experiments. The lower frame rate compared with the AGIPD system allows for a reduction in the equivalent noise down to 430 eV. Although only a 100 Hz frame is required for the SwissFEL, the Jungfrau system is designed for operation up to 2 kHz, making it also applicable for storage-ring based experiments. The same group at PSI is working on the Mönch system, which is a charge-integrating system with very low noise, 126 eV equivalent, and 25 µm pixels (Cartier *et al.*, 2014 ▶). These detectors show the potential of hybrid systems in the low-energy and small-pixel regimes normally reserved for monolithic systems. One of the most remarkable features will be the imaging of sub-pixel structures through photon-by-photon analysis of the photoabsorption positions.

### 3.3. Current trends in high-*Z* sensors   

**Figure 4:**
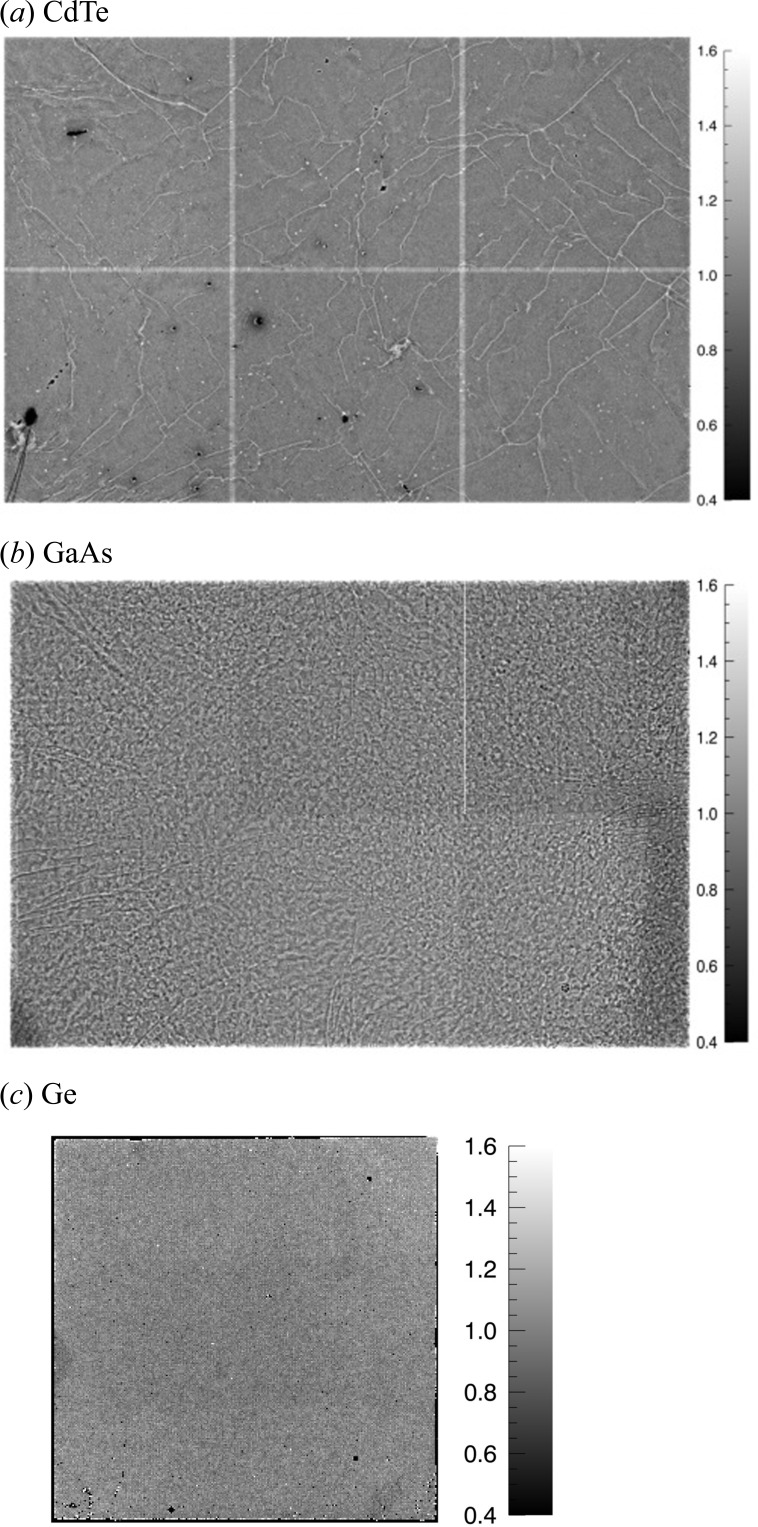
Flat-field images of high-*Z* sensors bump-bonded to 3 × 2 Medipix3 readout chips, taken using an X-ray source at 40 keV with an Mo target for sensors of (*a*) CdTe and (*b*) GaAs (Pennicard, Smoljanin *et al.*, 2014 ▶). The scales indicate the relative gain. (*c*) An image of a Ge sensor bump-bonded to a Medipix3 readout chip, taken using an X-ray source at 40 keV with an Ag target (Pennicard, Struth *et al.*, 2014 ▶). (Copyright SISSA Medialab Srl. Reproduced by permission of IOP Publishing. All rights reserved.)

Besides the developments in readout systems, some of which are described above, there has been very significant progress in the field of high-*Z* sensors. Silicon is a near-perfect sensor material for photon energies up to 15 keV, but its limited stopping power for higher-energy photons results in greatly reduced quantum efficiencies above 25 keV. In order to overcome this, groups around the world have been investigating alternative sensor materials, preferably with a wide-enough band gap to allow operation at room temperature. The markets for medical imaging and homeland security have been focusing on and heavily investing in CdTe and Cd(Zn)Te because of the very high *Z* of these materials, which is required to obtain sufficient stopping power for photons well above 100 keV (Koenig *et al.*, 2012 ▶). There has been steady progress in both material quality and processing capabilities over the years, and medium-sized sensors of a quality that is acceptable for certain synchrotron experiments are now available. Fig. 4 ▶(*a*) shows a flat-field image of a CdTe sensor mounted on 3 × 2 Medipix3 readout chips (Pennicard, Smoljanin *et al.*, 2014 ▶). The typical grain boundaries and growth imperfections associated with CdTe are clearly visible. These imperfections are generally not stable over time and with accumulated dose, so they can only partly be corrected by flat-field corrections. Another inconvenience of CdTe is the so-called polarization, related to the accumulation of charges at imperfections, which distorts the internal electric field. In order to overcome this, the bias on the sensor has to be switched off or even reversed periodically, which means interruption of measurements.

GaAs is an excellent candidate for photon energies up to 60 or 70 keV. A chromium-compensated material produced by Tomsk State University (Russian Federation) has recently shown very good performance and stability (Hamann *et al.*, 2015 ▶). Fig. 4 ▶(*b*) shows an uncorrected flat-field image of a GaAs:Cr sensor mounted on a Medipix3 readout system (Pennicard, Smoljanin *et al.*, 2014 ▶). Again, the typical line structure can be seen, which is related to material imperfections. Compared with CdTe, the raw image shows more inhomogeneity, but there are fewer insensitive pixels and the inhomogeneity is stable over time and accumulated dose. This means that effective flat-field correction is possible.

Ge is an alternative to GaAs for the medium photon energy range. It is a well known and highly perfect material available in large sizes. A disadvantage of Ge is the fact that it is highly sensitive to impurities and therefore difficult to process, and bump-bonding of the sensor to the readout chip has to be performed at low temperatures. A further complication is the small band gap, which requires the sensor to be cooled in order to reduce the leakage current. However, cooling to liquid nitrogen temperatures is not required, due to the small volume of the single pixels. Recently, DESY and the company CANBERRA jointly developed a pixelated sensor with 55 µm pixels, which was hybridized to a Medipix3 readout chip by the Fraunhofer Institute IZM in Berlin (Germany). The Medipix3 chip has a per-pixel leakage current compensation capability, and cooling the sensor to 173 K was sufficient for successful operation of the system (Pennicard, Struth *et al.*, 2014 ▶). A flat-field image is shown in Fig. 4 ▶(*c*). Except for a few unconnected pixels, the image shows excellent uniformity. The insensitive edge pixels arise due to the higher leakage current at the sensor edge; future production runs aim to reduce this. For certain high-end applications, the superior quality of Ge compared with GaAs is believed to outweigh the inconvenience of the cooling required. For example, in crystallography experiments, each Bragg peak may be concentrated on a few pixels, so uniformity of the detector is critical.

## 4. Monolithic detectors   

There are two pixel types for monolithic detectors, passive and active. Passive pixels have only a switching function that controls signal charge to flow to the periphery of the sensor. The most successful sensor with passive pixels is the CCD. Another example of passive pixels can be found in the XAMP (X-ray active matrix pixel) detector, with one switching transistor in a pixel that controls the flow of the signal charge (Chen *et al.*, 2002 ▶). On the other hand, active pixels have transistors for active functions such as amplification and processing. When active pixels are implemented in monolithic sensors, the sensor is sometimes called a monolithic active-pixel sensor (MAPS). We first discuss direct-detection CCDs as an example of passive-pixel detectors and summarize their current limitations. We then present selected on-going MAPS and related projects.

### 4.1. Passive-pixel detectors   

#### 4.1.1. Direct-detection CCDs   

Direct-detection CCDs offer several unique capabilities, such as low noise and a small pixel size of less than 20 µm. Another implementation of CCDs for X-ray imaging is indirect-detection CCDs, with phosphor as a converter of X-rays to optical photons. These have been a powerful tool (Gruner *et al.*, 2002 ▶). In this *Feature article*, we limit our discussion to direct-detection CCDs.

The CCD sensor has a charge-transfer structure on the entire image area. Each pixel has only two, three or four gates for the charge-transfer function. This simple pixel structure enables a small pixel size. The signal charge is transferred across the chip and converted to a voltage signal at the on-chip amplifier, such as a floating-diffusion amplifier (Figs. 1 ▶ *c* and 5 ▶ *a*). One of the advantages of this architecture resides at the amplifier: modulation of the internal potential transfers the signal electrons completely to a confined space (the charge-sensing node). It has a low enough capacitance that the voltage change associated with the transfer of a single electron can be as large as several microvolts. The CCD manufacturing process generally offers low-noise transistors for transmission of the voltage signal with minimum degradation of the noise. The total electronic noise is typically just a few electrons (one signal electron is generated by the absorption of a photon energy of 3.6 eV). Such low-noise performance enables not only X-ray intensity imaging, but also X-ray spectroscopic imaging. None of the hybrid detectors for X-ray imaging is reported to reach a noise floor of less than 10 electrons.

However, conventional scientific CCDs have several limitations in applications for advanced X-ray imaging. One of the shortcomings is the narrow thickness of the photodiode. Today, several CCD projects, some of which are discussed below, have achieved CCDs with a photodiode thickness greater than 300 µm, which corresponds to a quantum efficiency of 90% at 10 keV. Another weakness is the slow frame rate. Solutions for a higher frame rate vary from project to project. In the next section, we review several CCD projects.

#### 4.1.2. The pnCCD at the Max Planck Institute   

**Figure 5:**
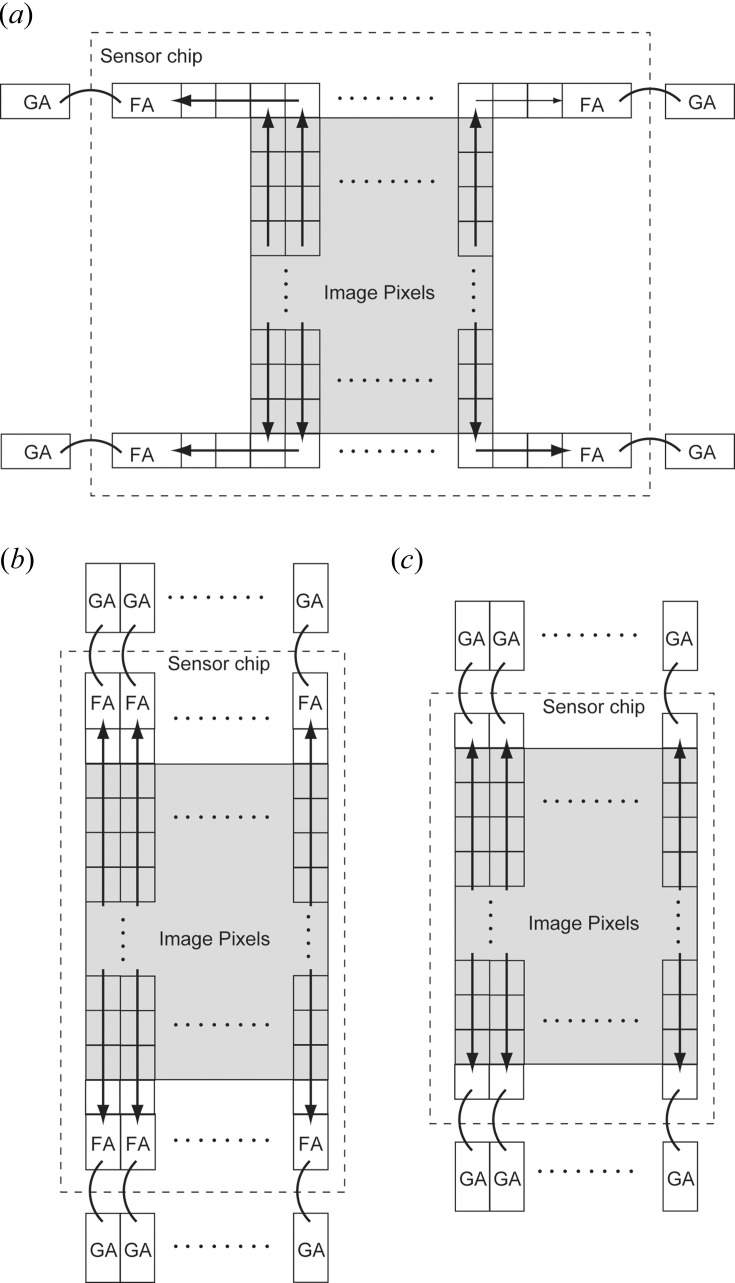
Architectures of CCD sensors with different degrees of parallel readout. (*A*) An example of a conventional scientific CCD with four readout ports. Each has a floating-diffusion amplifier (FA) wire-bonded to the external gain amplifier (GA). (*B*) A possible implementation of a fully column-parallel-readout CCD. (*C*) Fully column-parallel-readout CCD without floating-diffusion amplifiers, due to the small column pitch.

Conventional scientific CCDs run, at most, at 10 frames per second. The rate is slow because all the pixels are read in a serial fashion by a single or a few on-chip amplifiers (Fig. 5 ▶ *a*). Implementing multiple on-chip amplifiers can speed operations. The pnCCD developed by the Semiconductor Laboratory of the Max Planck Institute (Munich), with a pixel size of 75 µm, has an on-chip amplifier in one column similar to Fig. 5 ▶(*b*) (Strüder *et al.*, 2010 ▶). The output voltage waveform is transferred through wire bonding to CAMEX (complementary metal–oxide–semiconductor analogue multiplexing) signal processors and then to external 14-bit ADCs. The conversion of signal charge to voltage at the on-chip amplifiers proceeds in parallel to increase the frame rate. The system was operated at LCLS, with a maximum frame rate of 120 Hz synchronized to the LCLS pulses. During the first years of operation, the detectors installed in the CAMP chamber yielded important scientific results, such as serial femtosecond crystallography and coherent diffraction imaging (Seibert *et al.*, 2011 ▶; Chapman *et al.*, 2011 ▶). The detector system consists of two imaging planes, one for wider scattering and the other for smaller-angle scattering. Each of the imaging planes consists of two image sensors of 0.5 Mpixels. The low-noise performance of 2.5 electrons when operating at 223 K makes single-photon sensitivity possible through post-analysis of the images, even for very soft X-rays. For low-flux images, the photon energy was also resolved to distinguish the different X-ray fluorescence lines. A thick photodiode zone of 500 µm yielded good quantum efficiency, even for harder X-rays.

#### 4.1.3. The FastCCD at LBNL   

Another example is the FastCCD at the Lawrence Berkeley National Laboratory (LBNL; California, USA), with a pixel size of 30 µm (Denes *et al.*, 2009 ▶). In contrast with the pnCCD, FastCCD is based on an industry-standard metal–oxide–semiconductor (MOS) CCD structure, which in principle offers better manufacturability (Holland *et al.*, 2009 ▶). The column pitch of 30 µm is small, which means there is little space per column to implement the on-chip amplifier. Thus, the first FastCCD sensor had one amplifier per ten columns. Together with the dedicated development of the fCRIC (fast CCD readout integrated circuit) signal processor with built-in 15-bit floating-point readout with two 12-bit ADCs, these workers successfully developed detector systems operating at 200 frames per second, which have been used for ptychography CDI (coherent diffractive imaging), resonant inelastic X-ray scattering, XPCS and tomography on beamlines at the ALS (Advanced Light Source, Berkeley, California, USA), APS (Advanced Photon Source, Argonne, Illinois, USA) and LCLS (Doering, Chuang *et al.*, 2011 ▶). CCDs with a larger format of 1 Mpixel have also been developed with frame-store sections for an electronic shutter (Doering, Andresen *et al.*, 2011 ▶). Another variant of the 5 µm pitch CCD was also developed for X-ray spectroscopy imaging.

#### 4.1.4. MPCCD for SACLA at RIKEN   

**Figure 6:**
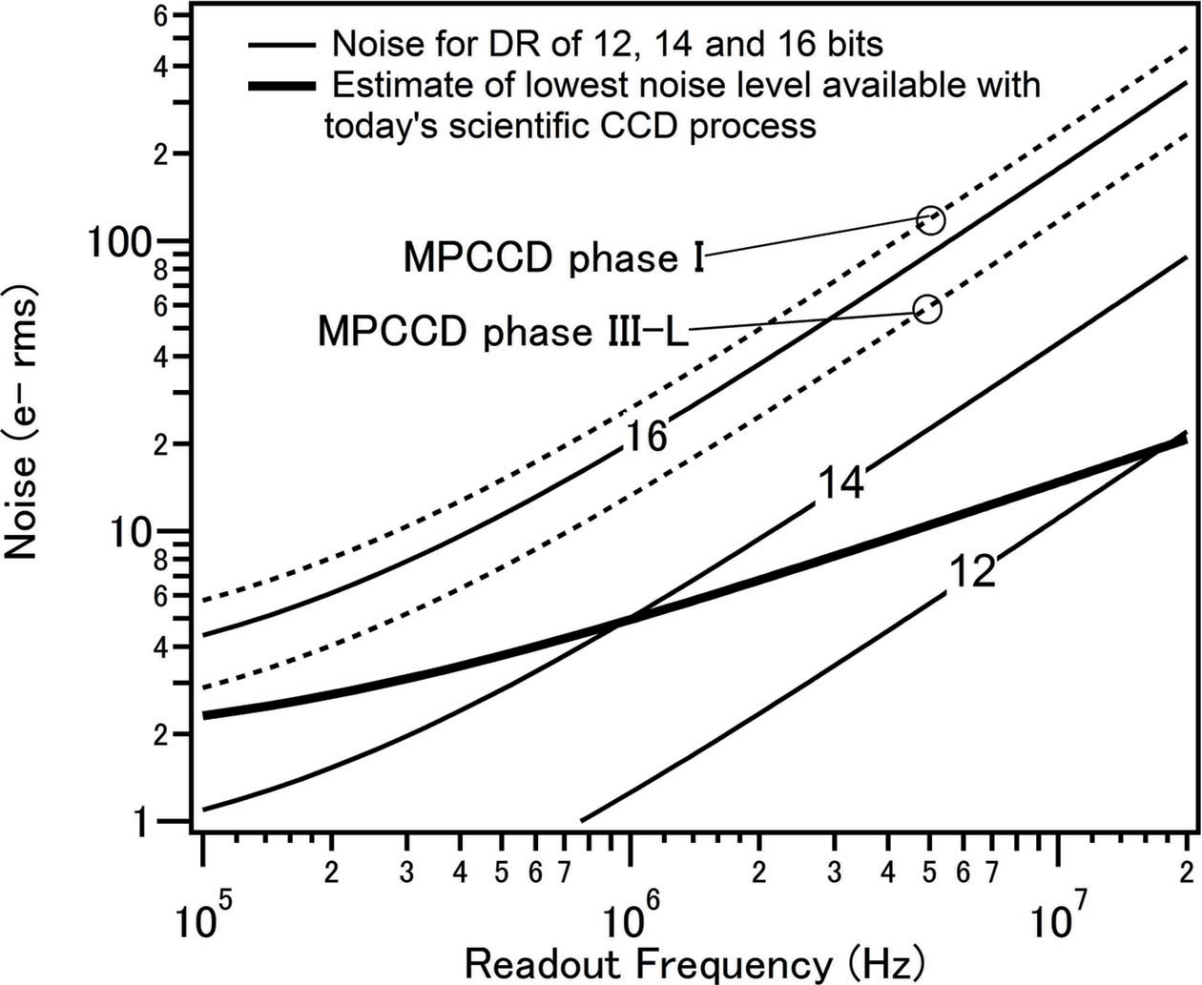
The noise performance obtainable with an optimum design approach of the floating-diffusion amplifier, plotted according to the equations of MPCCD design trade-offs reported in Appendix B of Kameshima *et al.* (2014 ▶). The solid curves indicate a trade-off with a constant dynamic range (DR = peak signal/electronic noise) of 12, 14 and 16 bits. As the readout frequency increases, the noise becomes worse. The bold solid line indicates an estimate of the noise level manufacturable using today’s scientific CCD processes. For a higher readout rate, the manufacturability limit becomes significant in terms of the achievable noise. The dashed curves represent the MPCCD phase I and phase III-L cases, with dynamic ranges of 15.4 and14.4 bits, respectively. Note that the noise values of the MPCCDs are larger by 2^1/2^ than standard CCDs due to their quasi-differential amplifier scheme, which enables robust operation through higher rejection of external noise.

**Figure 7:**
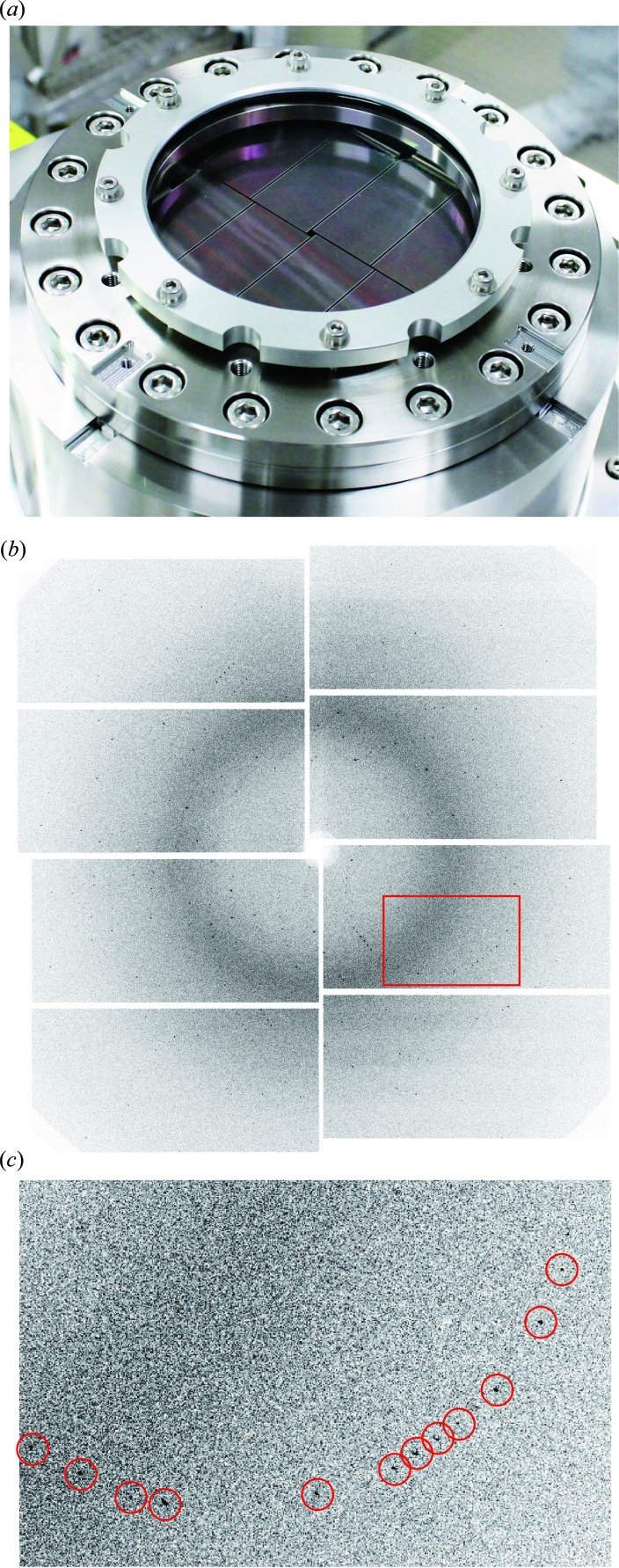
(*a*) The camera head of an MPCCD developed for serial femtosecond crystallography (Kameshima *et al.*, 2014 ▶). The detector consists of eight sensors aligned on a flat imaging surface to give a total of 4 Mpixels. The imaging surface can be placed close to the specimen to cover a scattering angle of ±45°. (*b*) A single-pulse diffraction pattern from a small lysozyme crystal recorded with this detector in combination with an early phase setup at SACLA (Song *et al.*, 2014 ▶). The data were calibrated by the facility’s standard automated procedure. Simple calibration is one advantage of passive pixels (Kameshima *et al.*, 2014 ▶). (*c*) An enlarged image of the area shown as a red rectangle in (*b*). Diffraction spots are indicated by red circles.

A different development direction of CCD technology can be found in multi-port CCDs (MPCCDs) for SACLA experiments (Kameshima *et al.*, 2014 ▶). With an emphasis on the peak signal, these workers have optimized MOS CCD technology towards a larger peak signal while keeping the frame rate of 60 Hz, the facility pulse frequency. The resulting pixel has a size of 50 µm, which is larger than typical MOS CCDs. Optimization to 6 keV X-rays resulted in a relaxed noise requirement and enabled a higher amplifier readout rate of 5 MHz (Fig. 6 ▶). A signal-to-noise ratio larger than 7 for 8 keV X-rays (100–250 electrons) was reported at 30 frames per second, matching the pulse frequency of user operations. One amplifier per 64 columns was implemented, resulting in eight on-chip amplifiers for a 0.5 Mpixel sensor. An image from early experiments on serial femtosecond crystallography is shown in Fig. 7 ▶, together with a camera head with 4 Mpixels. A dedicated dual-gain readout circuitry with two 16-bit ADCs gives a 19-bit floating readout, allowing the detection of a single photon to a peak signal of 2700 photons for 6 keV X-rays. The sensor has a 50 µm sensitive layer, which is too thin to detect X-rays above 10 keV. Recently, variants with 300 µm photodiode thickness have also been developed (Ono *et al.*, 2015 ▶).

#### 4.1.5. Other CCD applications and future possibilities   

Direct-detection MOS CCDs were also investigated for protein X-ray crystallography using synchrotron radiation by the Cornell group. The prototype sensor has a pixel size of 24 µm and a large imaging format of 4000 × 4000 pixels. Assessment results at the protein crystallography beamline F1 of CHESS (Cornell High-Energy Synchrotron Source, New York, USA) were reported by Green *et al.* (2013 ▶).

There are trade-offs between sensor parameters such as readout rate and noise. Current state-of-the-art on-chip amplifiers generate noise of only a few electrons at a readout rate of 1 Mpixel s^−1^ or less. The trade-offs for an optimum design approach of an on-chip floating-diffusion amplifier are shown in Fig. 6 ▶. A frame rate of the order of 1 kHz with 1 Mpixel, corresponding to a readout rate of 1 MHz, is thought to be the practical limit without degrading the noise level of about 5 electrons. To go beyond these trade-offs, new inventions such as an extremely parallel readout scheme with a pipeline architecture proposed by LBNL (Grace *et al.*, 2013 ▶) are required (Denes, 2014 ▶). The proposed scheme is presented in Fig. 5 ▶(*c*). A very fast CCD with a 50 µm pixel size targeting over 10 000 frames per second for LCLS II is reported to be developed by a collaboration between LBNL and SLAC (Blaj *et al.*, 2014 ▶).

A similar trade-off exists for the peak signal capacity. Scientific CCDs generally offer a maximum charge storage of 8000 electrons per 1 µm square (Janesick, 2001 ▶). An optimized pixel with 75% of its area used for charge storage will provide a peak signal density of 2.7 photons per 1 µm square for 8 keV X-rays. Peak signals hitherto reported in the literature have been limited to a few times less than this nominal limit, partly because of faster operation and design for manufacturability.

### 4.2. Monolithic active-pixel sensor (MAPS) projects for X-ray imaging   

In this section, we describe a few examples of MAPS development with some technical background. These implement either the latest sensor technologies and/or new inventions to overcome the trade-off limits inherent in CCDs. We try to link the technical aspects to their functions, and outline short-term performance improvements from the perspective of photon science.

#### 4.2.1. Femtopix and SOPHIAS based on silicon-on-insulator (SOI) pixel technology   

For hard X-ray imaging, a photodiode over 300 µm thick is required to maintain high quantum efficiency. The thick photodiode must be made of high-resistivity silicon and be biased by a high voltage. For high-quality imaging, floating-zone (FZ) wafers with minimum impurity concentration are used. Since they are mechanically fragile, dedicated treatment during production is mandatory. In contrast, sub-micron CMOS (complementary metal–oxide–semiconductor) transistors are made of low-resistivity Czochralski (CZ) silicon wafers. CZ wafers have a higher oxygen concentration, which results in higher mechanical strength than FZ wafers, and they are compatible with mechanically stressful short-time annealing, which gives the best performance for sub-micron CMOS transistors. Sub-micron CMOS transistors operate at just a few volts. In contrast, a photodiode as thick as 500 µm typically operates with a bias voltage of more than 50 V, and should be shielded against the sub-micron CMOS transistors. More than a decade of research based on commercially available foundry processes has still not yielded a feasible means of fabricating MAPS with thick photodiodes.

KEK, RIKEN and other institutes, mainly in Japan, are developing silicon-on-insulator (SOI) pixel technology as one of the methods enabling a combination of a thick photodiode and advanced CMOS transistors within a single monolithic chip (Arai *et al.*, 2010 ▶). This is realised by using custom SOI wafers, where the silicon layer for the CMOS, on top of a thin silicon oxide layer, is bonded to the wafer for the photodiode (Fig. 1 ▶ *d*). Additional fabrication steps are carried out to produce the metal pathways (vias) from the photodiode to the CMOS transistors through the thin silicon oxide layer, so that charge generated in the photodiode is transferred to the CMOS layer. The soft X-ray imager FemtoPix was developed by LBNL for femtoslicing experiments at ALS. In-pixel CMOS circuitry within a 17.5 µm pixel provides a fast gating function. The 192 × 192 pixel sensor operates at 4000 frames per second.

An additional advantage here is that we can use the photolithography technique at the sub-micron scale to tailor the photodiode implant structure. Signal-collecting electrodes with a spacing of a few microns can be produced. The SOPHIAS detector for the XFEL facility SACLA uses this feature to control the charge-collection step (Hatsui *et al.*, 2013 ▶). Most of the signal charge is transmitted to low-noise charge-collection electrodes. They form a low enough capacitance that a single X-ray photon of 6 keV is converted to a voltage of 4.7 mV. Such a high voltage swing can easily be read out, giving a low noise floor of 0.13 photons at 6 keV, but it will be saturated at 220 photons. The other electrodes collect only a portion of the charge and can measure up to 10 000 photons. The charge-division scheme, together with the in-pixel circuitry optimization, enables a high peak signal within a small pixel size of 30 µm. The charge-to-voltage conversion carried out without gain amplifiers makes the pixel very power efficient. The first system under development at SACLA will be used for coherent X-ray imaging of micron-scale objects. It consists of two sensors, comprising a total of 3.8 Mpixels at a frame rate of 60 Hz.

All the SOI pixel sensors described above are of the integrating type. Photon-counting pixels with SOI pixel technology have also been reported but are still in the experimental phase. In the case of SOI pixel technology, in-pixel transistors are located beneath the photodiode. They are physically very close to each other, with a separation of 0.2 µm (Fig. 1 ▶ *d*), so there is a higher chance of electric interference. A prototype MAMBO (monolithic active-pixel matrix with binary counters) sensor with a pixel size of 105 µm was reported by a group at Fermilab (Illinois, USA; Fahim *et al.*, 2013 ▶). They propose a nested well in the photodiode in order to shield it from the transistors to eliminate interference. Another shielding approach with a double SOI wafer has also been reported (Miyoshi *et al.*, 2013 ▶). We should note that the current SOI process is not yet radiation hard enough for utilization in photon energies above 7 keV, where X-rays are directly absorbed at the transistor layer (Kudo *et al.*, 2014 ▶).

#### 4.2.2. PERCIVAL imager for soft X-rays   

The performance figures for recent leading-edge CMOS image sensors are of particular interest as they are applicable to soft X-ray imaging. Noise performance close to one electron has been reported by several groups (Seitz & Theuwissen, 2011 ▶). The pixel size is reduced to nearly 1 µm for the imagers in mobile phones (Itonaga *et al.*, 2009 ▶). With an industry-leading production line with 300 mm wafers, a chip size of 20.2 × 20.2 cm has been reported (Yamashita *et al.*, 2011 ▶). These technologies together create a good opportunity to collect all the desired functions within a single detector system. Historically, these high-end CMOS image sensors have been produced only on the in-house production lines of commercial companies, inaccessible to the academic community. However, in recent years several semiconductor companies have offered foundry services for CMOS image-sensor processing.

The PERCIVAL (pixellated energy-resolving CMOS imager, versatile and large) project is the first where an academic development programme has taken the opportunity of designing a scientific X-ray imager using a commercial CMOS image-sensor foundry (Wunderer *et al.*, 2014 ▶). The collaboration team from DESY, Rutherford Appleton Laboratory (RAL; Didcot, UK), Elettra (Trieste, Italy) and Diamond Light Source (DLS; Didcot, UK) are now developing a sensor with a 27 µm pixel size and an imaging area of 14 × 14 cm that runs at 120 frames per second for soft X-rays in the range 250–1000 eV. The project envisages versatile soft X-ray applications in both synchrotron and FEL sources. To realise their target peak signal of 10^5^ photons at 500 eV, nearly two orders of magnitude higher than optical imaging demands, a threefold LOFIC (lateral overflow integration capacitor) is proposed. The LOFIC was originally reported for optical imaging as a powerful technique to achieve a high dynamic range (Wang *et al.*, 2001 ▶; Akahane *et al.*, 2009 ▶). PERCIVAL has a global electronic shutter: the leakage current is accumulated only for the duration of the exposure. For FEL experiments, where the exposure time can be very short, it may provide an opportunity for cryostat-free operation. This would greatly enhance the versatility of the detector setup, especially in soft X-ray experiments that require optical paths under ultra-high vacuum.

In order to detect soft X-rays below 400 eV, where the attenuation length is about 100 nm, the sensors are back-illuminated. The entrance window, where the incoming soft X-rays are absorbed and not detected, should be thinner than the attenuation length to achieve a high quantum efficiency. The processing method for the backside of consumer optical CMOS image sensors typically results in the formation of an entrance window with a thickness of the order of 100 nm. Therefore, these cannot provide optimum quantum efficiency for soft X-rays. The formation of a defect-free entrance surface and a very shallow electrode on the backside is mandatory. In contrast with CCDs and the sensors for hybrid detectors, where high-temperature annealing can be adopted (Holland *et al.*, 2009 ▶), backside processing for CMOS image-sensors in practice demands a low-temperature process to prevent damage to the aluminium wiring layer formed in the preceding process steps. Therefore, a soft X-ray MAPS requires dedicated development for the backside process. The PERCIVAL project aims to address this issue with a goal of 90% quantum efficiency for the 250–1000 eV range by introducing a dedicated backside process.

The present approach based on commercial CMOS image-sensor processing may be extended to higher photon energies in the near future by making the photodiode layer thicker. In this context, preliminary results from the LePix project were reported by Giubilato *et al.* (2013 ▶). A few CMOS image-sensor foundries are already providing photodiodes with thicknesses of 30–50 µm. Industrial needs for near-infrared imagers will continue to drive development towards ever thicker photodiodes. As X-rays become harder, photons start to penetrate the photodiode and hit the CMOS transistors. In the case of a silicon photodiode with a thickness of 500 µm, the penetration occurs above a photon energy of 7 keV. X-ray radiation hardness of CMOS transistors becomes essential. Some promising evaluation results for X-ray radiation hardness have been reported from the perspective of high-energy physics and space applications (Goiffon *et al.*, 2010 ▶; Abelev, 2014 ▶). The peak signal will also be improved in the near future. Today’s CMOS process can provide a higher capacitance density than those used in the projects discussed so far. The peak signal density of the PERCIVAL approach can potentially be increased by more than one order of magnitude.

#### 4.2.3. Non-linear DEPFET sensors for DSSC   

In the DSSC detector under development discussed as a hybrid detector in §3.2.2, the sensor for the DSSC is connected to the readout electronics chip through bump bonding (Porro *et al.*, 2012 ▶). The DEPFET device on the sensor acts as part of an amplifier offering non-linear conversion, which enables low noise and high peak signal detection, and can be considered as a MAPS as well. From this perspective, the DSSC detector can be considered the first hybridization project of MAPS for X-ray imaging, and has the advantages of both hybrid and monolithic detectors, namely complex signal processing inside the pixel and low noise, respectively. The concept of a DEPFET with nonlinear conversion at the charge-conversion step will be of interest for a wide range of X-ray imaging applications, especially in high-speed imaging where lossless information compression offers the advantage of reduced data size.

#### 4.2.4. Three-dimensional integration   

An advantage of a monolithic sensor is low-noise performance at lower power dissipation. The physical reason for this is the low capacitance between the photodiode and the readout circuitry. The capacitance can be reduced in hybrid detectors if the interconnect is small enough. Fermilab, Brookhaven National Laboratory (New York, USA) and AGH University of Science and Technology (Kraków, Poland) are collaborating to develop the VIPIC (vertically integrated photon-imaging chip) detector for X-ray correlation spectroscopy (Deptuch *et al.*, 2010 ▶). They have hybridized the sensor and the readout chip using a three-dimensional integration technology with an oxide–oxide fusion bonding that features tiny connections of a few microns (Fig. 1 ▶ *e*). This three-dimensional integration technology offering low-noise performance at lower power dissipation will be of interest in photon science.

A group from Fermilab and Argonne National Laboratory (Illinois, USA) is developing a FASPAX (Fermi–Argonne large-area pixel array for X-ray detection) detector (Deptuch *et al.*, 2014 ▶). It has charge-integration pixels using a bipolar current splitter in order to achieve a high peak signal. They use three-dimensional integration technology for wafer-scale detector integration. The device also has analogue storage inside the pixels to capture an image every 150 ns, similar to the detectors for the European XFEL.

### 4.3. Practical aspects of MAPS production   

We need to address the practical trade-offs between performance and manufacturability. The cost of constructing and operating the latest semiconductor production line far exceeds the capacity of most research budgets. If the differences between the sought-after chip and consumer chips reside only in design (*e.g.* the size and type of transistors and their location, the wiring pattern *etc.*), then production can be outsourced to semiconductor companies offering foundry services. In this way, the scientific community has benefitted from advances in semiconductor technology with a delay of about ten years. This is the case for the readout chips for hybrid detectors. Another example can be found in signal processors for MAPS. A signal-processing chip for very fast CCDs uses 65 nm CMOS technology to process the signals at a very high speed with lower power dissipation (Grace *et al.*, 2013 ▶). The 65 nm technology is more advanced than the 130 nm technology which is generally used by academic groups developing X-ray imaging detectors, and it generally offers a higher power efficiency and higher data rates of digital outputs.

The features of MAPS also depend on the semiconductor processes. Performance figures, such as frame rates, are improved with later technology even with an identical design strategy. However, MAPS for X-ray imaging currently requires customization of the production. Since the latest infrastructures exist only in factories operated by large corporations, changing the production conditions is more difficult for the small-volume production of scientific X-ray imagers. The choice of production infrastructure is therefore a critical decision in every MAPS project. For example, in the case of the non-linear DEPFET, elaborate process optimization is carried out at a small factory (Porro *et al.*, 2012 ▶). SOPHIAS endeavours to develop a new type of sensor with minimum modifications to the process for consumer products (Arai *et al.*, 2010 ▶; Hatsui *et al.*, 2013 ▶). In the case of PERCIVAL, the use of dedicated equipment at an academic laboratory for optimum backside electrode formation was chosen after completion of all the CMOS processes at a commercial factory, so that the two steps do not interfere with each other (Wunderer *et al.*, 2014 ▶).

## 5. Summary   

We have discussed research and development programmes for X-ray imaging detectors with a focus on hybrid and monolithic detectors. Hybrid detectors with counting pixels have gained popularity in the synchrotron radiation community. Today, a number of development projects currently underway aim to extend their performance. The rise of XFEL facilities demands the integrating type of pixels because photons arriving within less than 100 fs cannot be distinguished, even by state-of-the-art detector technology. The XFEL image sensors are developed using both hybrid and monolithic technologies. One of the most challenging light sources from the perspective of detector development is the European XFEL, which has a burst pulse train at a high repetition rate of 4.5 MHz. The LPD, DSSC and AGIPD projects addressing this issue have been presented in some detail. For some soft X-ray imaging detectors, CCD technology has been used since it offers low noise, indispensable for the detection of soft X-ray photons. CCD technology has been extending its range of applications to XFEL and synchrotron radiation experiments in the hard X-ray regime. PERCIVAL, a MAPS project, recently reported its first results for a prototype. It takes full advantage of commercial CMOS image-sensor processing, which was not accessible ten years ago. The project will also address other challenges, such as backside processing that is compatible with this process. Another development goal is to achieve MAPS with a very thick photodiode for hard X-ray applications. SOI pixel-sensor technology has a thick photodiode and CMOS active circuitry combined on a single chip, minimizing drawbacks by placing a silicon dioxide layer in between. A few sensors have been developed using this process. Intensive development of three-dimensional integration technology is also in progress. Among other advantages of three-dimensional integration in photon science, developments towards lower noise and wafer-scale integration have been addressed. In this *Feature article* we have not discussed digital data transfer, on-the fly analysis, visualization, archiving or post analysis. As detectors produce ever more data, these digital frameworks will become more important in future.
